# Bioinformatics analysis of potential pathogenesis and risk genes of immunoinflammation-promoted renal injury in severe COVID-19

**DOI:** 10.3389/fimmu.2022.950076

**Published:** 2022-08-16

**Authors:** Zhimin Chen, Caiming Chen, Fengbin Chen, Ruilong Lan, Guo Lin, Yanfang Xu

**Affiliations:** ^1^ Department of Nephrology, Blood Purification Research Center, The First Affiliated Hospital, Fujian Medical University, Fuzhou, China; ^2^ Research Center for Metabolic Chronic Kidney Disease, The First Affiliated Hospital, Fujian Medical University, Fuzhou, China; ^3^ Department of Traditional Chinese Medicine, The First Affiliated Hospital, Fujian Medical University, Fuzhou, China; ^4^ Central Laboratory, The First Affiliated Hospital, Fujian Medical University, Fuzhou, China; ^5^ Department of Intensive Care Unit, The First Affiliated Hospital, Fujian Medical University, Fuzhou, China

**Keywords:** COVID-19, renal injury, WGCNA, nomogram, bioinformatics, STEM

## Abstract

Renal injury secondary to COVID-19 is an important factor for the poor prognosis of COVID-19 patients. The pathogenesis of renal injury caused by aberrant immune inflammatory of COVID-19 remains unclear. In this study, a total of 166 samples from 4 peripheral blood transcriptomic datasets of COVID-19 patients were integrated. By using the weighted gene co-expression network (WGCNA) algorithm, we identified key genes for mild, moderate, and severe COVID-19. Subsequently, taking these genes as input genes, we performed Short Time-series Expression Miner (STEM) analysis in a time consecutive ischemia-reperfusion injury (IRI) -kidney dataset to identify genes associated with renal injury in COVID-19. The results showed that only in severe COVID-19 there exist a small group of genes associated with the progression of renal injury. Gene enrichment analysis revealed that these genes are involved in extensive immune inflammation and cell death-related pathways. A further protein-protein interaction (PPI) network analysis screened 15 PPI-hub genes: *ALOX5*, *CD38*, *GSF3R*, *LGR*, *RPR1*, *HCK, ITGAX, LYN, MAPK3, NCF4, SELP, SPI1, WAS, TLR2* and *TLR4*. Single-cell sequencing analysis indicated that PPI-hub genes were mainly distributed in neutrophils, macrophages, and dendritic cells. Intercellular ligand-receptor analysis characterized the activated ligand-receptors between these immune cells and parenchyma cells in depth. And KEGG enrichment analysis revealed that viral protein interaction with cytokine and cytokine receptor, necroptosis, and Toll-like receptor signaling pathway may be potentially essential for immune cell infiltration leading to COVID-19 renal injury. Finally, we validated the expression pattern of PPI-hub genes in an independent data set by random forest. In addition, we found that the high expression of these genes was correlated with a low glomerular filtration rate. Including them as risk genes in lasso regression, we constructed a Nomogram model for predicting severe COVID-19. In conclusion, our study explores the pathogenesis of renal injury promoted by immunoinflammatory in severe COVID-19 and extends the clinical utility of its key genes.

## 1 Introduction

The World Health Organization (WHO) formally designated the disease caused by the severe acute respiratory syndrome coronavirus 2 (SARS-CoV-2) as coronavirus disease 2019 (COVID-19) on February 11, 2020 (1). Over 70% of severe COVID-19 patients with basic disease progress swiftly to acute respiratory distress syndrome, metabolic acidosis, or multi-organ failure (2). Accumulating evidences suggested aberrant activation of immune cells, elevated plasma cytokine concentrations (IL-6, IL-10, TNF, and others), and cytokine storm are significant causes of SARS-CoV-2 infected individuals with multi-organ failure (3). Epidemiological studies suggest that acute or chronic kidney injury has a substantial effect on patients’ prognoses (4). The reported incidence of AKI in patients infected with COVID-19 varies from 0.5 to 28.0% (5, 6). According to a recent study, 43.0% to 59.0% of patients presented with varying degrees of proteinuria on admission, and 26.7% to 44.0% of patients presented with hematuria (7, 8). Furthermore, anomalies in renal imaging were observed, including increased perirenal fat density, perirenal fascial thickening, and interstitial fluid (9). In addition to the deleterious effects of SARS-CoV-2 that impairs the antiviral immune response and binds directly to the angiotensin-converting enzyme 2 (*ACE2*) receptor in the kidney (10–13). Risk factors such as hemodynamic abnormalities, mechanical ventilation, and antibiotic therapy also all contribute significantly to the development of COVID-19 renal injury (4, 14). The truth is that the imbalance of immune homeostasis caused by such multifactorial causes is the most important driver of COVID-19 progression (15). Due to the limitations of traditional experimental methods and the availability of appropriate samples, it is difficult to investigate the effects of such complicated immune status on the kidney in depth.

Recently, an increasing amount of medical research has shifted its focus to the molecular and genetic levels of disease, and network-based strategies have allowed a more in-depth examination of disease mechanisms (16). Nashiry MA et al. (17). innovatively employed bioinformatics to investigate the association between COVID-19 and digestive and cardiovascular diseases. It has opened up a new avenue for exploring the pathophysiology of COVID-19-associated renal injury. In this study, we performed a comprehensive bioinformatic analysis of single-cell transcriptome data from our mouse kidney injury model and the transcriptome data of COVID-19 and kidney injury from public databases. We aimed to explore the pathogenesis and risk genes of immunoinflammation-promoted COVID-19 renal injury.

## 2 Materials and methods

### 2.1 Data download and pre-processing

Transcriptome datasets are accessed through the GEO database (https://www.ncbi.nlm.nih.gov/geo/) of the National Center for Biotechnology Information (NCBI). Search the database for “COVID-19”, “blood”, and “PBMC” to obtain high throughput sequencing datasets related to COVID-19. A total of 4 COVID-19 datasets were included as a discovery set. The GSE196822 dataset is a peripheral blood transcriptome data of COVID-19 from India, containing 9 healthy individuals, 8 mild COVID-19 patients, 10 moderate COVID-19 patients, and 7 severe COVID-19 patients. The GSE179627 dataset is peripheral blood mononuclear cell (PBMC) transcriptome data of COVID-19 and contains data from 26 healthy individuals, 3 patients with mild COVID-19, and 10 patients with moderate COVID-19. The GSE171110 dataset is a peripheral blood transcriptome data of COVID-19, containing 10 healthy individuals, and 44 patients with severe COVID-19. The GSE197204 dataset contains 42 severe COVID-19 peripheral blood transcriptomic data. The GSE157103 dataset was used as an independent validation set, for sample selection, see sections 2.8 and 2.9. All patients with COVID-19 in the dataset were confirmed positive by PCR, and the classification criteria for mild, moderate, and severe COVID-19 among different datasets were classified according to international guidelines for the severity of COVID-19 (18–24). GSE98622 (25) is a dataset of renal ischemia-reperfusion injury (IRI) which contains 18 normal mouse kidneys and 10 consecutive time points of IRI kidney injury data, with 3 samples for each time point. The original matrix was normalized by log_2_ transformation after missing values were replenished with the “impute” R package. In the case of a single gene corresponding to multiple expressions, the average value was taken as its gene expression. Four different provenanced datasets in the COVID-19 discovery set were integrated using the “inSilicoMerging” R package (26). The “ComBat” function of the “sva “R package was used to remove batch effects (27).

### 2.2 Weighted gene co-expression network analysis

The “WGCNA” R package (28) was used to create a weighted gene co-expression network to identify key modules and genes in the discovery COVID-19 set. First of all, the “hcluster” function was used to cluster and eliminate outliers to maintain the network’s stability. After that, the optimal soft threshold with scale-free topology fit index (R^2^) > 0.80 and good average network connectivity is selected using the “pickSoftThreshold” function and the network is converted to a scale-free network. The “adjacency” function is applied to convert to a topological overlap matrix. Subsequently, hierarchical clustering was performed using the dynamic shear tree method, resulting in an overall clustering tree of COVID-19 differential genes. By iteratively clustering the eigenvector genes of different modules, modules with high similarity can be obtained, thus constructing a weighted co-expression network of differentially expressed genes. Correlations between gene modules and COVID-19 phenotypes were calculated. Positive correlation modules with p-values less than 0.05 were considered key modules associated with the phenotype and included in the subsequent analysis. Genes within the key modules were further screened by calculating module membership (MM) and gene significance (GS) values; We set |MM|>0.8 and |GS|>0.1 to filter COVID-19 key genes following the official WGCNA guidelines (28) and previous application examples (29, 30) to obtain the most relevant genes to the traits in the key modules.

### 2.3 Short time series expression miner analysis

To identify the gene expression pattern of COVID-19 renal injury, we performed STEM analysis (31, 32), which can cluster and analyze the expression pattern of time-series expression data sampled chronologically. Each gene was assigned to the closest trend by calculating the Pearson correlation distance between genes and the predicted expression trend in the expression profile. The expected number of genes assigned to each trend was computed using the exhaustive method of permutations, and the significance level of genes within the trend was calculated. The parameters were set as follows: 1) Maximum Unit Change in model profiles between time points is 1; 2) Maximum output profiles number is 5 (similar profiles will be merged); 3) Minimum ratio of the fold change of differentially expressed genes is no less than 2.0.

### 2.4 Gene enrichment and protein-protein interaction network analysis

The Gene Ontology (GO), Kyoto Encyclopedia of Genes and Genomes (KEGG), and Reactome enrichment analysis was conducted through the “clusterProfiler” R package (33) and the results were visualized through the “ggplot2” R package (34). The STRING database (https://string-db.org/) was used to perform PPI network analysis on key genes of COVID-19 renal injury with a minimum effective binding score of 0.4. Cytoscape 3.7.2 software (https://cytoscape.org/) was used to visualize the results of the PPI network analysis. The degree values of nodes in the PPI network are calculated using the cytoHubba (35) plugin in the Cytoscape. A higher degree value implies that the highly regarded nodes play a significant part in the network’s topology, and these genes are defined as PPI-hub genes.

### 2.5 Construction of IRI-AKI mouse model and Single-cell transcriptome sequencing

C57BL/6 mice (8–10 weeks, 23–26 g, male) were obtained from the pathogen-free (SPF) facility of Fujian Medical University. All animal experiments were approved by the Laboratory Animal Management and Ethics Committee of Fujian Medical University and were performed following the “China Guide for the Protection and Use of Laboratory Animals”. All mice were housed in a specific pathogen-free facility with a 12-hour light/dark cycle. In the procedure of IRI-AKI surgery, retroperitoneal clipping surgery was used. After mice were anesthetized with ketamine (80-100 mg/kg/i.p., Cayman Chemical) and xylazine (10 mg/kg/i.p., Selleck Chemicals), approximate 10-mm incisions were performed at a distance of about 8 mm on each side of the spine. After both kidneys were carefully exposed, bilateral renal pedicles were clamped for 43 min by a vascular clip (Fine Science Tools). The kidney was observed visual with a loss of blood supply and turning pale. After the vascular clamps were removed to restore the blood supply with visually reperfusion, the surgical incisions were closed in two layers with 5-0 sutures. The mice were then injected with pre-warmed physiological saline solution (37°C; 1 ml per 20g body weight) subcutaneously (s.c.). At the end of these procedures, mice were put back in cages in a temperature-controlled room (25°C) where free access to water and food was available. Cell capture was performed using the official library kit (10X Genomics Chromium Single-Cell 3’ kit, V3) according to the manufacturer’s instructions. Following the capture of target cells, sequencing was performed using the NovaSeq6000 sequencing platform (paired-end multiplexing run, 150 bp) by LC-Bio Technology Co. Ltd. (Hangzhou, China).

### 2.6 Single-cell transcriptome sequencing data analysis

GSE163668 (36) is a single-cell transcriptome dataset of COVID-19 peripheral blood which contains samples from different states of COVID-19 patients. Both the single-cell data from IRI-AKI and COVID-19 were analyzed through the “Seurat” procedure (37). In IRI-AKI, the following parameters were used to remove low-quality cells: (1) exclude cells expressing ≤500 or ≥4,000 genes/cell; (2) exclude cells expressing ≤500 or ≥15,000 unique molecular identifier per cell (UMIs/cell); (3) exclude cells with high cell complexity (log10GenesPerUMI) ≤0.8; (4)since renal tubular epithelial cells in the kidney are very energetically involved in active transport, which requires a large number of mitochondria to provide energy (38, 39); we exclude cells with >30% mitochondrial ratio; (5) exclude doublets using the “DoubletFinder” (Version 2.0.3) package; (6) retain genes expressed in at least 10 cells. The quality control steps in COVID-19 PBMC were the same as the original study (36). The LogNormalize method of the “Normalization” function was used for expression homogenization. The “FindVariableGenesfunction” function selected 2,000 highly variable genes based on the average expression and dispersion of each gene. Since cell cycle arrest is a normal manifestation of AKI pathology, we did not regress the effect of cell cycle genes on the results. The “harmony” algorithm (40) was then used to minimize the batch variation and merge the data, and the “FindClusters” function determines the proper resolution and clusters all cells. We use the “RuntSNE” function to reduce the dimensionality, and available biomarkers labelled the clustered cells to identify cell types. Finally, PPI-hub genes were visualized in severe COVID-19 blood and IRI-AKI kidneys using the “FeaturePlot” function.

### 2.7 Ligand-receptor interaction analysis between renal immune- parenchymal cells

We used “CellPhoneDB” (Version 2.1.7) (41) to investigate potential ligand-receptor interactions between renal immune and parenchymal cells. A total of 18,130 homologous genes were obtained after human-mouse homologous gene conversion using the “bioMart” (Version 2.46.3) package. We first randomly arranged cluster markers for all cells 1,000 times to determine the average receptor and ligand expression levels of the interacting clusters. This produced a zero distribution for each receptor-ligand pair. *P*-values for the cell type-specific likelihood of the corresponding receptor-ligand complex were obtained by calculating the proportion of means above the actual mean and the data were visualized by weighted network plots. Chemokines and immunostimulatory pathway-related ligand-receptors were selected for analysis and the intercellular interaction weight network was plotted. The greater the degree of intercellular interaction, the thicker the lines in the network and the larger the corresponding interaction numbers.

### 2.8 Construction of a random forest model by using PPI-hub genes in an independent validation set and clinical data analysis in Nephroseq database

The R package “randomForest” was used to construct a random forest model to validate the expression of PPI-hub genes in an independent COVID-19 dataset: GSE157103. Severe COVID-19 patients (n=50) and non-severe COVID-19 patients (n=50) in this dataset were considered as outcome variables, and 15 PPI-hub genes were used as response variables. The train and test sets are divided in the ratio of 7:3. The train set is used for modeling with 3000 trees and 15 variables to randomly select each tree, and the test set is used to evaluate the accuracy of the model. To find the optimal mtry parameter (i.e., the optimal number of variables in the binomial tree of the specified node), we performed recursive random forest classification on all possible numbers in the variables and calculated the average error rate of the model. After randomly sampling the training set with put-back for the random forest, the variables are ranked by the relative importance of the final obtained response variables. The importance was assessed by the degree of mean decrease accuracy and the degree of mean decrease Gini index. At last, the accuracy of the model is evaluated in the test set by the receiver operating characteristic curve (ROC) and visualized using the “pROC” R package. Subsequently, we verified the expression of PPI-hub genes, and the significance test was performed using the Wilcoxon-Mann-Whitney test, and the results were visualized using the “ggplot2” R package. In addition, we analyzed the association between PPI-hub genes and clinical features through the Nephroseq database (http://v5.nephroseq.org/). Following the calculation of Pearson correlation coefficients between co-DEGs and glomerular filtration rates (GFRs), a scatter plot was constructed.

### 2.9 Lasso regression to screen variables and construct Nomogram prediction model

To further establish a prediction model capable of predicting the development of COVID-19 patients to severe status, COVID-19 patients admitted to the ICU were considered as the severe COVID-19 group and the remaining COVID-19 patients were considered as the Control group. We evaluated the clinical trait data corresponding to the sample, traits with more than 40% missing values were excluded (apacheii scores, lactate, and sofa scores were excluded). Samples with missing values for the remaining traits were also excluded. Finally, 78 samples with complete data on CRP, D-Dimer, procalcitonin, fibrinogen, Charles score, ferritin, age, and gender were included in the lasso regression. The median expression levels of PPI-hub genes were utilized as cut-off values for classifying genes into high- and low-expression categories, which were considered as risk genes and then combined in lasso regression analysis. The “glmnet” R package was used to perform lasso regression. Lasso regression is a sophisticated algorithm for variable selection in multicollinear or high-dimensional data. Previous research has established that lasso regression simplifies the model’s complexity and enhances its prediction accuracy (42). After including the screening risk variables into the prediction model, by using the “rms” R package, we constructed a nomogram prediction model that can predict COVID-19 patients progressed to severe status. The clinical validity of the model was determined using the ROC analysis, calibration curve, C index, and decision curve analysis (DCA).

## 3 Results

### 3.1 WGCNA identifies key genes for mild, moderate and severe COVID-19

**Figure 1:**
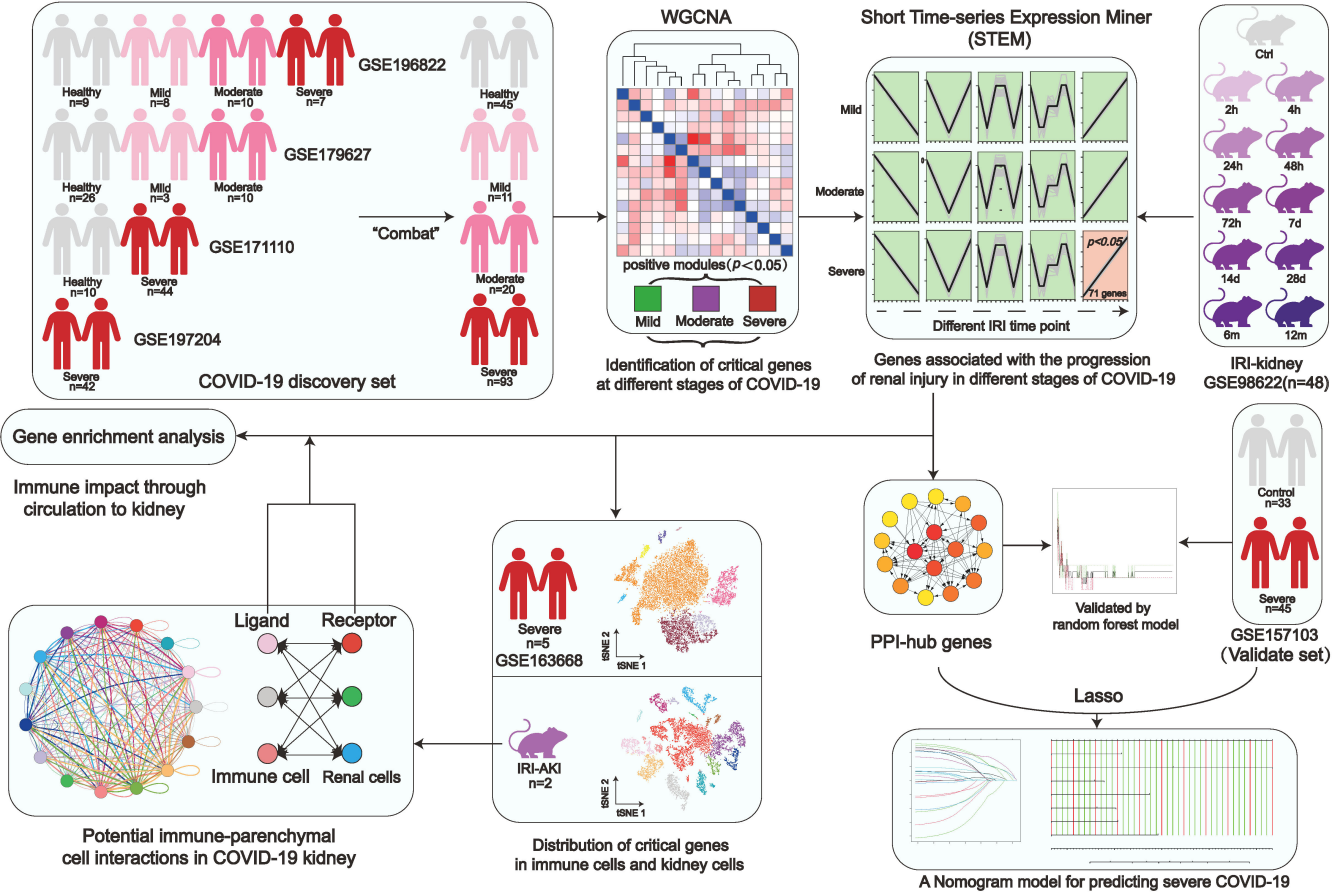
Study workflow. Coronavirus disease 2019, (COVID-19); Weighted gene co-expression network analysis, (WGCNA); Ischemia-reperfusion injury, (IRI); Kyoto encyclopedia of genes and genomes, (KEGG); Protein-Protein interaction, (PPI).

**Figure 2:**
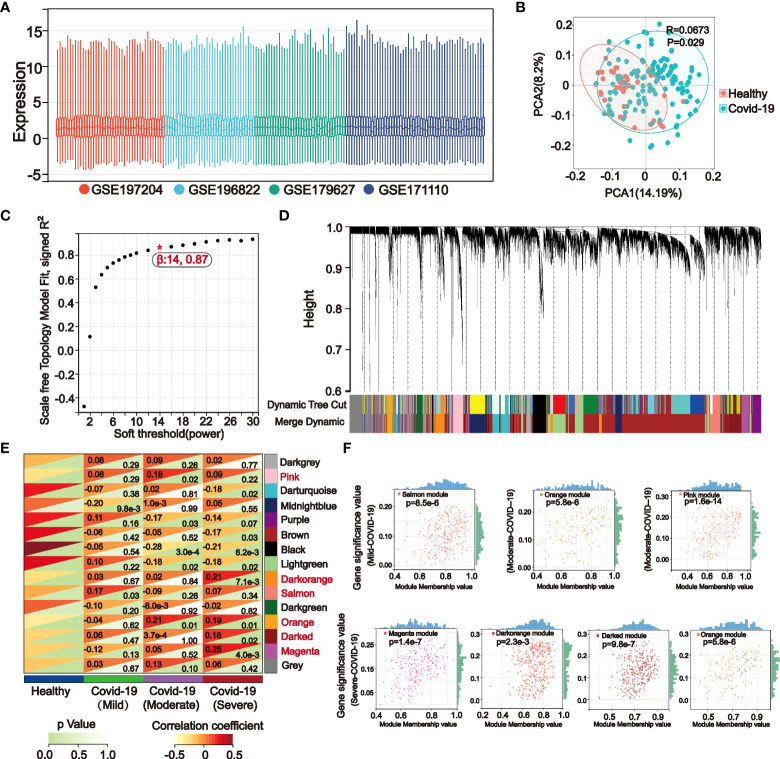
The weighted gene co-expression network (WGCNA) identifies key genes in mild, moderate, and severe COVID-19 modules. **(A)** The boxline plot after integrating the data suggests that batch effects between different datasets were removed. **(B)** Principal component analysis (PCA) of the integrated dataset showed significant differences between the Healthy and COVID-19 samples (*p*<0.05). **(C)** WGCNA analysis was performed on the integrated COVID-19 dataset, and the topological overlap matrix was constructed by calculating the optimal soft threshold. **(D)** Similar gene modules were merged in WGCNA by the dynamic shearing tree method. **(E)** Gene modules-clinical features correlation heatmap. WGCNA calculated correlation coefficients and *p*-values between clinical features and 15 gene modules, of which the gray modules were nonsignificant. Modules with the *p*-values < 0.05 in the mild (Salmon), moderate (Pink, Orange), and severe (Magenta, Darked, Orange, Darkorange) COVID-19 were selected for subsequent analysis. **(F)** Correlation dotplots of gene significance (GS) value and module membership (MM) value for the selected key modules in mild, moderate, and severe COVID-19, |MM|>0.8 and |GS|>0.15 were set to filter COVID-19 key genes.

A flow chart was created for the whole experiment to illustrate the details of the data more explicitly (**Figure 1**). A total of 166 samples were included in the discovery set of COVID-19 after integrating the four datasets. Sample boxline plots showed that batch effects from different datasets were removed (**Figure 2A**) and principal component analysis (PCA) showed that there were significant differences between the healthy and COVID-19 samples (*p*<0.05) (**Figure 2B**). The different status of COVID-19 samples was included as clinical traits in the WGCNA analysis. By combining both genetic and clinical trait data, the gene expression profile of complex biological processes can be divided into several highly correlated signature modules to identify genes of interest. For constructing the scale-free topological overlap matrix, “pickSoftThreshold” function selects β=14 as the optimal soft threshold β (**Figure 2C**). Identification and merging of similar gene modules were performed by the dynamic shearing tree method. The shearing height was set to 0.25, and the minimum number of genes in each module was set to 50. A total of 15 gene modules were identified, of which the gray modules were nonsense modules (**Figure 2D**). The modules with *p*-value <0.05 were selected as the key modules for each of the COVID-19 mild, moderate, and severe traits. This led to the identification of the salmon module (*R*=0.17, *p*=0.03) as a key module for mild COVID-19, orange (*R*=0.21, *p*=0.01) and pink (*R*=0.18, *p*=0.02) module as key modules for moderate COVID-19, and in severe COVID-19, magenta (*R*=0.25, *p*=0.004), darkorange (*R*=0.21, *p*=0.007), orange (*R*=0.19, *p*=0.01) and darked (*R*=0.18, *p*=0.02) module were identified as a key modules (**Figure 2E**). Subsequently, |MM| >0.8 and |GS| >0.1 were set to screen key genes in each module (**Figure 2F**, **Supplementary Table S1**). Gene enrichment analysis of key genes suggested that progressive severe immune and coagulation-related events were activated during COVID-19 progression, which also validated the reliability of key genes (**Supplementary Figure S1**).

### 3.2 STEM analysis indicates that severe COVID-19 is associated with the progression of kidney injury

**Figure 3:**
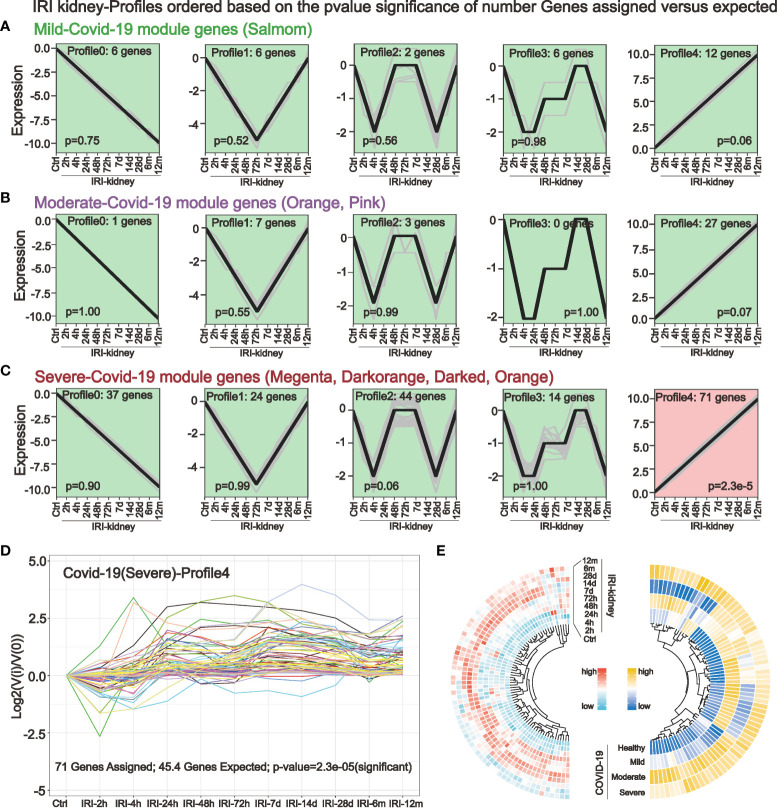
Short-time sequence expression miner (STEM) identifies genes associated with the progression of kidney injury in different COVID-19 stages. **(A–C)** STEM analysis was performed in a dataset with consecutive time points of IRI kidney injury by using key genes of mild **(A)** moderate **(B)** and severe **(C)** COVID-19. Five different gene expression profiles were simulated to identify genes that are altered in parallel with COVID-19 in the IRI kidney. **(D, E)** STEM analysis revealed that only in severe COVID-19, a small cluster of key genes was associated with IRI kidney injury **(D)**. These 71 genes had the same expression trend and were elevated after 24h of IRI, with some genes persistently highly expressed in the subsequent IRI time points **(E)**, these genes are potentially highly correlated with COVID-19 renal injury.

STEM analysis can identify a group of differentially expressed genes with the same expression pattern in a continuous-time point expression profile. In a dataset with 10 consecutive time points of IRI kidney injury, we performed STEM analysis by using mild (**Figure 3A**), moderate (**Figure 3B**), and severe (**Figure 3C**) COVID-19 pathogenesis key genes obtained in WGCNA as input genes. The STEM algorithm simulated 5 representative gene expression trends with time progression in the original IRI kidney injury expression profile in advance. These 5 trends were considered as the trajectories of gene changes associated with the progression of kidney injury. Then the algorithm assigned the input genes to the 5 trends for clustering and differential expression analysis. The results indicated that only in severe COVID-19 there existed 71 genes with significantly differential synchronous changes (**Figures 3C, D** **)**. The validation of expression also showed that in the kidney, the expression of these 71 genes gradually elevated after 24h of IRI, and part of them persistently highly expressed up to 12 months of IRI. And in COVID-19 peripheral blood, these 71 genes were highly expressed in severe COVID-19 (**Figure 3E**).

### 3.3 Gene enrichment analysis of COVID-19 kidney injury-related genes and identification of PPI-hub genes

**Figure 4:**
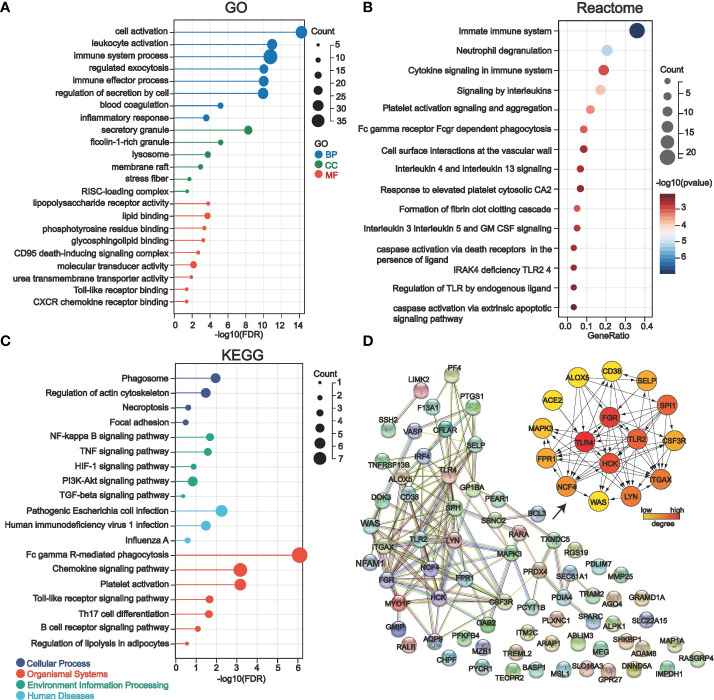
Gene enrichment analysis and protein-protein interaction network (PPI) analysis of 71 key genes in severe COVID-19 renal injury. **(A–C)** GO **(A)**, Reactome **(B)**, and KEGG **(C)** enrichment analysis of COVID-19 renal injury-related genes. **(D)** The PPI network of COVID-19 renal injury-related genes was constructed by the STRING database, and the hub genes in the network were further screened by the cytohubba plugin of cytoscape software.

We deduced the specific functions and signaling pathways of the key genes of COVID-19 kidney injury *via* GO, Reactome, and KEGG enrichment analysis. The results show that the main GO entries enriched included various immune cell responses and cell death-related processes **(** **Figure 4A** **)**. The entries enriched by Reactome analysis include multiple transcription factor pathways associated with the immune response **(** **Figure 4B** **)**. The KEGG pathways involved include various cellular stress pathways associated with inflammation and cell death receptors, as well as pathways associated with viral infection **(** **Figure 4C** **).** Our STRING database results showed the protein interaction associations of the COVID-19 kidney key genes; we imported the results into cytoscape software to calculate the degree values inside the networks. Fifteen of the 71 genes (*ALOX5*, *CD38*, *GSF3R*, *LGR*, *RPR1*, *HCK*, *ITGAX*, *LYN*, *MAPK3*, *NCF4*, *SELP*, *SPI1*, *WAS*, *TLR2* and *TLR4*) interacted with other genes and have higher degree values which are hub genes in the PPI network (**Figure 4D**). Notably, *ACE2*, a known critical target of COVID-19 infecting renal cells, was engaged in the PPI network by interacting with *TLR4*.

### 3.4 Single-cell sequencing reveals the distribution of PPI-hub genes in immune cells and renal parenchymal cells

**Figure 5:**
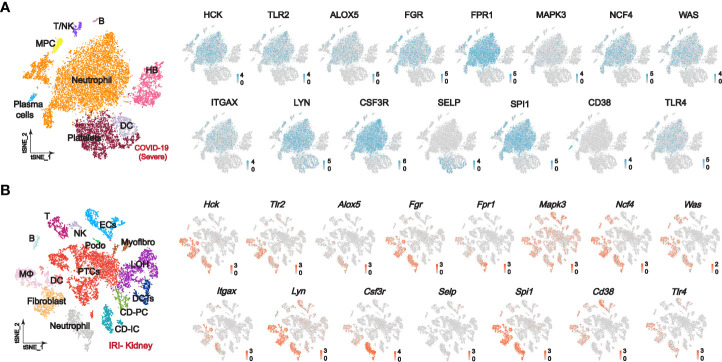
Single-cell sequencing explores the expression of PPI-hub genes in immune and kidney cells. **(A)** In the peripheral blood of patients with severe COVID-19, the PPI-hub genes were predominantly expressed in neutrophils, macrophages, and dendritic cells. **(B)** In renal parenchymal cells of IRI-AKI mice, PPI-hub genes were mainly expressed in proximal tubular cells, podocytes, and endothelial cells. In IRI-AKI renal immune cells, PPI-hub genes were mainly expressed in neutrophils, macrophages, and dendritic cells.

We further explored the distribution of PPI-hub genes in immune cells and renal cells by single-cell sequencing. Notably, since COVID-19-induced immune storm and tissue ischemia-reperfusion injury is an etiology of pre-nephrotic kidney injury, STEM analysis also implicates overexpression of PPI-hub gene correlates with acute kidney injury in severe COVID-19 patients. Therefore, we constructed a classic pre-nephrotic kidney injury model: the IRI-AKI mouse model to simulate this process. The single-cell sequencing of peripheral blood from a public database of patients with severe COVID-19 suggested that the PPI-hub genes were predominantly distributed in neutrophils, macrophages, and dendritic cells (**Figure 5A**). Renal single-cell sequencing of IRI-AKI showed that in renal parenchymal cells, the PPI-hub genes were predominantly distributed in proximal tubular cells (PTCs), podocytes, and endothelial cells (ECs). And similar to peripheral blood in severe COVID-19, these genes were also predominantly distributed in neutrophils, macrophages, and dendritic cells in renal immune cells (**Figure 5B**).

### 3.5 Ligand-receptor analysis reveals potentially activated immunoinflammatory pathways in COVID-19 Kidneys

**Figure 6:**
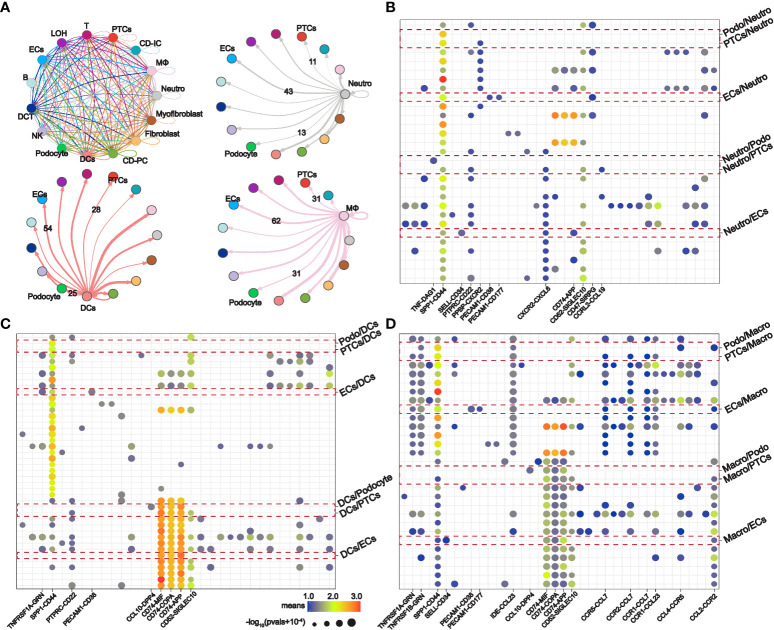
Intercellular ligand-receptor analysis explores potential renal immune cell-parenchymal cell interactions in COVID-19 kidney injury. **(A)** Ligand-receptor interaction network between specific renal immune cells (neutrophils, macrophages, and dendritic cells) and parenchymal cells (proximal tubule cells, podocytes, and endothelial cells). **(B–D)** Expression of immunostimulatory and chemokine-related ligand receptors between specific renal immune cells and parenchymal cells.

**Figure 7:**
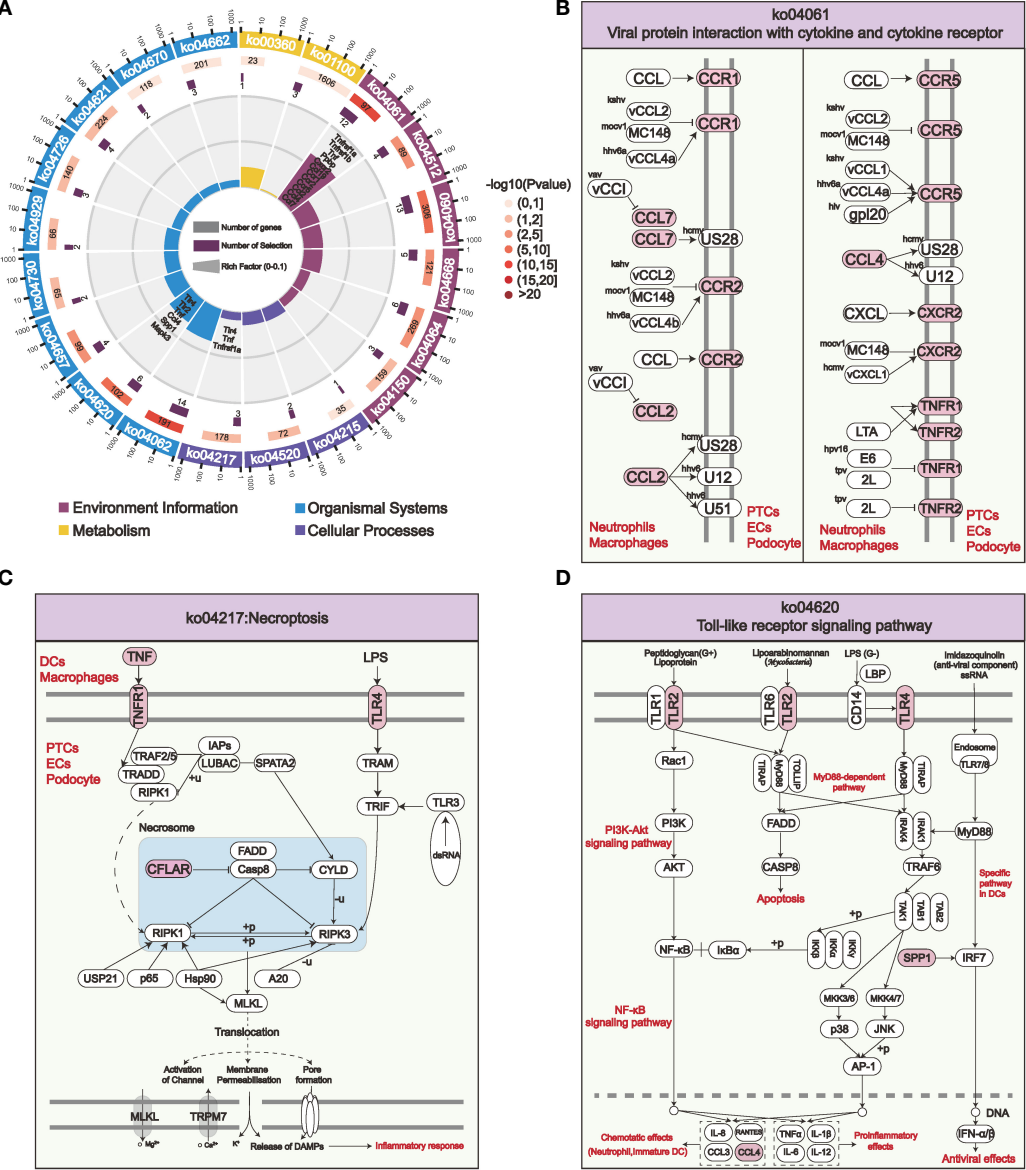
KEGG enrichment analysis of the PPI-hub genes and activated intercellular immunostimulatory and chemokine-related ligand receptors for COVID-19 kidney injury. **(A)** The most significantly enriched pathways in the four major classes of KEGG are shown. **(B–D)** Viral protein interaction with cytokine and cytokine receptor **(B)**, necroptosis **(C)**, and Toll-like receptor signaling pathways **(D)** are activated among specific immune cells and parenchymal cells in the kidney, these pathways may be crucial for COVID-19 renal injury.

STEM analysis indicated that the persistent overexpression of PPI-hub genes during renal injury may be associated with AKI progression. Moreover, it is well recognized that immune cell infiltration plays an important role in the progression of kidney injury. Therefore, in combination with the distribution of PPI-hub genes, we performed ligand-receptor analysis between immune cells and parenchymal cells in IRI-AKI data by “CellPhoneDB”. In AKI kidneys, there exist extensive intercellular ligand-receptor interactions between immune and parenchymal cells. We highlighted the interaction network between cells where PPI-hub genes are predominantly distributed: neutrophils, macrophages, dendritic cells and proximal renal tubular cells, podocytes, and endothelial cells (**Figure 6A**). Then, dotted heatmaps present a more detailed illustration of the specific interaction of intercellular chemokine-related ligand receptors and immunostimulatory pathway-related ligand receptors (**Figures 6B–D**). These ligand receptors may be critical molecules in the progression of renal injury due to immune cell infiltration in COVID-19. All these genes (red dashed line) were incorporated into the KEGG pathway analysis along with the PPI-hub genes to further explore the signaling pathways activated between renal immune cells and parenchymal cells (**Figure 7A**). We found that viral protein interaction with cytokine and cytokine receptors (**Figure 7B**), necroptosis (**Figure 7C**), and Toll-like receptor signaling pathway (**Figure 7D**) emerged more prominently in the results. Combined with their actual biological functions, we proposed that they may be key pathways in COVID-19 renal injury.

### 3.6 PPI-hub genes of COVID-19 renal injury can be used to predict the occurrence of severe COVID-19

**Figure 8:**
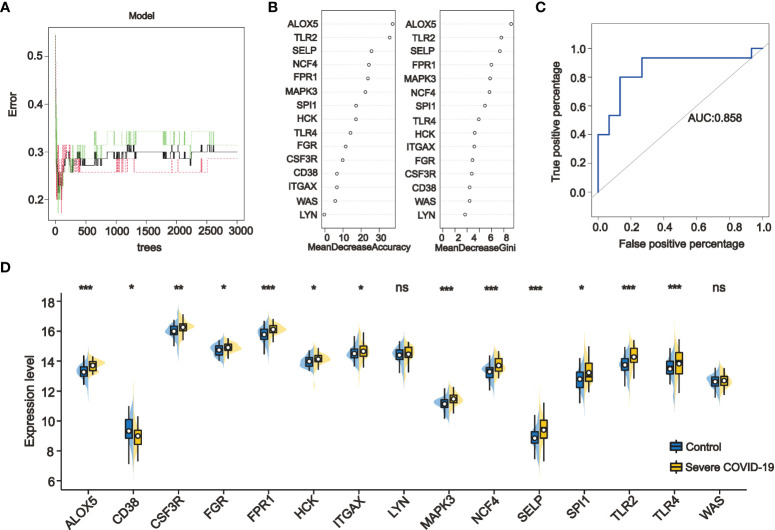
Ranking the importance of PPI-hub genes and validating their diagnostic efficacy by random forest. **(A)** By iteratively optimizing the parameters, 3000 decision trees are ultimately selected to construct the random forest model. **(B)** The importance of the variables was ranked by mean decrease accuracy and mean decrease Gini, with higher values indicating that the variable contributes greater to the accuracy of the model. **(C)** The accuracy of the random forest model was validated in the test set, and the results showed that the model had a high accuracy (AUC: 0.858). **(D)** Validation the expression of PPI-hub genes in an independent COVID-19 dataset, most of the genes were significantly upregulated in patients with severe COVID-19 (*p < 0.05, **p < 0.01, ***p < 0.001, ns, not statistically significant).

**Figure 9:**
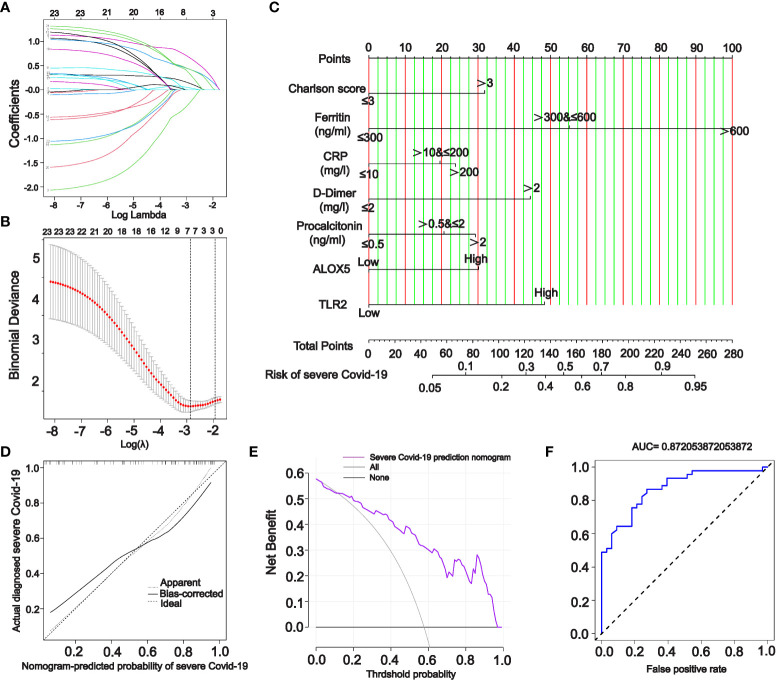
A nomogram model for predicting severe COVID-19. **(A, B)** Incorporation of PPI-hub genes and clinical characteristics data together in lasso regression for screening variables in an independent COVID-19 dataset. **(C)** Using screened variables to construct a nomogram model for predicting severe COVID-19. **(D–F)** Calibration curves **(D)**, DCA curves **(E)** and ROC curves **(F)** all indicate that the nomogram model is highly accurate and possesses good diagnostic performance.

For ranking the importance of PPI-hub genes and validating their diagnostic efficacy. We built a random forest classifier using PPI-hub genes as response variables in an independent COVID-19 dataset. The optimal mtry value for random forest selection after optimized parameters is 2 and the number of decision trees is 3000 (**Figure 8A**). The ranking of variables’ importance determined by the mean decrease accuracy index and mean decrease Gini index showed that *ALOX5*, *TLR2*, *SELP*, *FPR1*, *MAPK3*, *NCF4* (MeanDecreaseGini>6) had a greater contribution to the accuracy of the model (**Figure 8B**). The accuracy of the random forest model was validated in the test set, and the results showed that the model had high accuracy (AUC: 0.858) (**Figure 8C**). In addition, we verified the expression pattern of PPI-hub genes through statistical tests. The results showed that most of the genes, as in the discovery set (except *CD38*, *LYN*, and *ITGAX*) (**Figure 3E**), were significantly upregulated in patients with severe COVID-19 (*p* < 0.05) (**Figure 8D**). Subsequently, PPI-hub genes were correlated with clinical traits of kidney disease through the Nephroseq database. The results showed that all PPI-hub genes were associated with low GFR in kidney disease (**Supplementary Figure S2**). And finally, based on the clinical characteristic information provided by the raw data of the validation set, Fifteen PPI-hub genes combined with 8 clinical traits were included in lasso regression to screen for variables. The results showed that the coefficients of the influences initially included in the model were compressed as the penalty coefficient λ changed. Some of the factors were compressed to zero. The λ value at the cross-validation error of λmin + ambda.1se was selected as the optimal value of the model, and 8 variables were finally screened out, which were Charlson score, ferritin, CRP, D-Dimer, procalcitonin, *ALOX5* and *TLR2* (**Figures 9A, B** **)**. Based on these risk factors, we established a nomogram to predict whether patients with COVID-19 would progress to the severe status (**Figure 9C**). The nomogram model enables calculating a score for each patient predictor, and the sum of these values is used to get the overall score. The total score related to the predictive value represents the risk probability of the COVID-19 patient developing a severe condition. The model was evaluated, and the calculated C-index was 0.872, the area under the ROC curve was 0.872, and both the calibration curve and DCA analysis indicated that the model had a favorable outcome (**Figures 9D–F**).

## 4 Discussion

Previous studies on COVID-19 renal injury focused more on changes caused by the virus binding directly to *ACE2* in the kidney (4, 43–45). However, kidney injury promoted by persistent system-wide immunoinflammation has been neglected. In this study, we used comprehensive bioinformatics approaches to identify genes involved in the progression of kidney injury in severe COVID-19. In the renal microenvironment, these genes may contribute to the progression of renal injury by facilitating the activation of immunoinflammatory-related pathways through abundant ligand-receptor interactions between immune-parenchymal cells. And considering these genes as risk genes for COVID-19 renal injury could better predict the occurrence of severe COVID-19.

We characterized key genes of COVID-19 in mild, moderate to severe in this study. Similar results were obtained previously by Hasankhani et al. who characterized COVID-19 expression profiles usin who characterized COVID-19 expression profiles using WGCNA (46). As COVID-19 progresses, the expression of hub genes changes subsequently with the activation of an increasing number of immunoinflammatory-related functions and pathways. The progressive intensification of the cytokine storm throughout the system and the cascade activation of platelets leads to coagulation disorders and the reduction of tissue oxygenation. This process is gradually being revealed (10, 43, 47, 48). One of the limitations of our study, the lack of complete age and gender data in the integrated dataset due to the limitations of integrating multiple datasets, may have implications for several putative pathways.

Our findings highlight the role of immune cells from the circulation infiltrating into the kidney of patients with severe COVID-19 through 15 hub genes. *ALOX5* was the gene that contributed most to the accuracy of the random forest model in the validation set. This gene encodes a member of the lipoxygenase gene family that plays a dual role in the synthesis of leukotrienes from arachidonic acid (49). In addition to inflammatory processes, *ALOX5* is also involved in dendritic cell migration, wound healing, and adhesion to endothelial cells *via ITGAM* and *ITGAX* on monocytes (50, 51). It is well known that dysfunction of peritubular capillary endothelial cells is a recognized cause of ischemia and hypoxia in PTCs during the pathogenesis of AKI (43, 52, 53). Thus, this gene may be a critical molecule causing endothelial cell injury in COVID-19 kidneys. The identified PPI-hub genes also include three tyrosine-protein kinase family members: *HCK*, *FGR*, and *LYN*. These genes can deliver signals from cell surface receptors and play important roles in the regulation of innate and adaptive immune responses, integrin signaling, and responses to DNA damage and genotoxic agents (54–58). In myeloid and B-lymphocyte lineage cells, *HCK* may help a couple of the Fc receptors to the activation of the respiratory burst (59), which may promote the formation of cytokine storms in COVID-19. In addition, HCK can phosphorylate WAS and participate in extracellular stimulation of cytoskeleton remodeling, phagocytosis, cell adhesion, and migration together with *FGR* and *LYN* (60, 61). Thereby, it regulates gene transcription and repair of damaged DNA, promotes mast cell degranulation, and releases inflammatory cytokines. This may be one of the essential mechanisms of immune cell activation in COVID-19.

The proteins encoded by *TLR2* and *TLR4* can form heterodimers with other TLR family members to recognize conserved molecules of microorganisms and play an important role in pathogen recognition and innate immune activation (62, 63). Previous studies have shown that dimerization of caspase-8 and Toll-like receptors 2 and 4 can trigger the activation of NLRP3 inflammatory vesicles in human monocytes (64, 65). Subsequently, Lyn/Syk-dependent calcium entry and reactive oxygen species production are activated, leading to the activation of caspase-8. In the humanized mouse model, such cascade activation of *TLR2*, *TLR4*, and *LYN* triggers multiple inflammation-associated cell death pathways, such as the formation of necrosome in necroptosis, which activates human monocytes to impede endothelial regeneration and promote kidney injury (66, 67). Moreover, in COVID-19, dysregulation of necrosome is an important mechanism that promotes cytokine storm and intense immune inflammatory events (68, 69). Vaccine adjuvants targeting Toll-like receptor agonists are also considered promising therapeutic targets (70, 71). *FPR1* gene encodes a G protein-coupled receptor for mammalian phagocytes that mediates the phagocytic response to microbial invasion of the host and plays an important role in host defense and inflammation (72, 73). *MAPK3* encodes proteins that act in signaling cascade responses to regulate various cellular processes such as proliferation, differentiation and cell cycle progression in response to various extracellular signals (74, 75). Previous studies on COVID-19 have suggested that *FPR1* and *MAPK3* may be potential therapeutic drug targets (54, 76). In addition, our study also highlights the abnormal activation of platelets in severe COVID-19. SELP, a protein that redistributes to the plasma membrane during platelet activation and degranulation, and mediates the interaction of activated endothelial cells or platelets with leukocytes (77–79). Among the PPI-hub genes, we also characterized several key genes that have not been studied in depth. *NCF4*, which encodes NADPH oxidase in phagocytes (80, 81). *CSF3R*, which controls the production and differentiation of granulocytes (82, 83). *SPI1*, a transcriptional activator that may be specifically involved in the differentiation or activation of macrophages or B cells (84, 85). Further studies are needed in the future to explore the role of these genes in COVID-19 renal injury.

Along with the risk genes screened in lasso regression, Charlson score, ferritin, D-Dimer, CRP, and procalcitonin are screened as risk factors for severe COVID-19. CRP and procalcitonin are often used markers of inflammation in humans, particularly in the setting of severe infections or inflammatory responses. A Meta-analysis by Lippi G et al. who characterized COVID-19 expression profiles usin (86) also suggested that elevated PCT levels increased the risk of conversion to severe COVID-19 by nearly 5-fold [OR=4.76, 95% CI (2.74, 8.29)]. Single-center retrospective research indicated that the level of CRP was positively correlated with the severity of COVID-19 disease (87). Tyurin et al. also indicated that systemic disruption of the immune system in COVID-19 can lead to defects in adaptive immune cell subsets and elevated CRP levels, subsequently contributing to COVID-19 progression (88). In addition, researchers observed many erythrocyte aggregates blocking the capillary lumen at autopsy (89), as well as sporadic iron-containing heme particles in the renal tubular epithelium of COVID-19 patients. These findings imply that renal vascular obstruction may be a critical factor in the development of renal injury. The renin-AngII system can cause microvascular damage and accelerate the progression of acute tubular necrosis and cortical necrosis, potentially leading to irreversible renal failure (90, 91).

We believe these findings will contribute to the understanding of the pathogenesis of immune inflammation-promoted kidney injury in COVID-19. Early detection of kidney injury in COVID-19 and potentially arrest of progression of severe COVID-19 can be achieved by detecting and intervening in these risk genes. Due to the limitations of this study, more rigorous *in vivo* and *in vitro* experiments are needed in the future to substantiate these findings.

## 5 Conclusion

Our study identified 15 risk genes associated with the progression of kidney injury in severe COVID-19: *ALOX5*, *CD38*, *GSF3R*, *LGR*, *RPR1*, *HCK, ITGAX, LYN, MAPK3, NCF4, SELP, SPI1, WAS, TLR2* and *TLR4*. In the peripheral blood, these genes are predominantly expressed in a variety of inflammatory immune cells and may alter the immune microenvironment of the kidney with circulation. In the kidney, these genes may potentially promote the progression of renal injury through extensive ligand-receptor interactions between immune-parenchymal cells, activating multiple immune-inflammatory-related pathways including viral protein interaction with cytokine and cytokine receptor, necroptosis, and Toll-like receptor pathways. These findings may contribute novel insights into the pathogenesis of COVID-19 kidney injury. In combination with the nomogram model which includes risk genes suggest that early detection and intervention of these genes in the clinic may be helpful in the treatment of severe COVID-19.

## Data availability statement

The datasets presented in this study can be found in online repositories. The names of the repository/repositories and accession number(s) can be found in the article/Supplementary Material. The mouse IRI-renal single cell sequencing can be found in the NCBI GEO repository, accession number GSE197266.

## Ethics statement

The animal study was reviewed and approved by the First Affiliated Hospital, Fujian Medical University.

## Author contributions

ZC and YX designed the study. ZC, CC, FC, RL, GL and YX performed data analysis and data interpretation. ZC, CC, and FC conducted the bioinformatics and statistical analyses. ZC and YX wrote the original draft. YX supervised the research and performed writing-review and editing. All authors read and approved the final paper.

## Funding

This work was supported by grants from National Natural Science Foundation of China (No.82070720), Fujian Provincial Health Technology Project (No.2021ZQNZD004), Natural Science Foundation of Fujian province (No.2020J02020), and Fujian Province Finance Project (2020B009).

## Acknowledgments

YX was supported by the plan of Young Experts with Outstanding Contribution to Health in Fujian Province, and Outstanding Young Talents Program of First Affiliated Hospital of Fujian Medical University (YJCQN-XYF2021).

## Conflict of interest

The authors declare that the research was conducted in the absence of any commercial or financial relationships that could be construed as a potential conflict of interest.

## Publisher’s note

All claims expressed in this article are solely those of the authors and do not necessarily represent those of their affiliated organizations, or those of the publisher, the editors and the reviewers. Any product that may be evaluated in this article, or claim that may be made by its manufacturer, is not guaranteed or endorsed by the publisher.
