# Supplementary material for: Bioinformatics analysis of potential pathogenesis and risk genes of immunoinflammation-promoted renal injury in severe COVID-19
Source: Front Immunol. 2022 Aug 16;13:950076. doi: 10.3389/fimmu.2022.950076 (PMC9424635; doi:10.3389/fimmu.2022.950076)
Supplement: Supplementary file 1 [file DataSheet_1.docx]

Supplementary Material

**Supplementary Table 1.** The key genes of mild, moderate and severe COVID-19 screened by WGCNA

| **Mild COVID-19** | **Moderate COVID-19** | **Severe COVID-19** |
| --- | --- | --- |
| *ABHD8, ADAP1, ADRM1, ARAF, ARHGDIA, CHMP1A, COPE, CORO1A, CORO7, COX8A, CYBA, DAPK3, ESRRA, FBXL15, JOSD2, KLF16, MAP1S, MAP2K2, MFSD10, MRPL28, NR1H2, OGFR, PYCARD, RAB1B, RBM42, RNPEPL1, RPUSD1, SCAF1, SCYL1, TBC1D10B, TMEM115, ZDHHC12, ZNF524*  **(Salmon)** | *ABLIM3, ALOX12, ARHGAP6, C1orf198, CTDSPL, CTTN, EGF, EHD3, ELOVL7, ESAM, F13A1, GP1BA, GP6, ITGB3, ITGB5, MAP1A, MGLL, PCSK6, PCYT1B, PDLIM1, PEAR1, PF4, RHOBTB1, SELP, SPARC, TSPAN9, TUBB1* **(Orange)**  *ADAR, APOL6, BST2, CCR1, CMPK2, CMTR1, DDX60, DHX58, DTX3L, EIF2AK2, EPSTI1, GBP1, GRAMD1B, GTPBP2, HELZ2, HERC5, HERC6, HESX1, IFI16, IFI35, IFI44, IFI44L, IFI6, IFIT1, IFIT2, IFIT3, IFIT5, IRF7, IRF9, KIAA1958, LAMP3, LAP3, MOB3C, MOV10, MX1, MX2, OAS1, OAS2, OAS3, OASL, PARP12, PARP14, PARP9, PLSCR1, PML, RNF213, RSAD2, RTP4, SAMD9, SAMD9L, SERPING1, SNX20, SP100, SP110, SPATS2L, STAT1, STAT2, TNFSF10, TOR1B, TRIM56, UBE2L6, USP18, XAF1, ZBP1, ZCCHC2, ZNFX1* **(Pink)** | *ACSL1, AGO4, ALPK1, AQP9, B4GALT5, BCL6, CFLAR, CR1, DENND5A, FCGR2A, GAB2, GPR27, KCNJ15, KCNJ2, LYN, MANSC1, MAPK14, MCTP2, MEGF9, MSL1, MTMR3, MXD1, NAMPT, NRBF2, NUMB, PLXNC1, PPP4R1, RALB, RBM47, REPS2, RNF149, RNF24, SIPA1L2, SLC22A15, SRPK1, SSH2, STX3, TGFA, TLR2, TLR4, TMCC3, USP32, VNN2, WDFY3* **(Meganta)**  *AATK, ADAM8, AGTRAP, ALOX5, ARAP1, BASP1, BCL3, CDA, CSF3R, DGAT2, DHX34, DOK3, DYSF, ECE1, EHD1, FAM53C, FGR, FLOT2, FMNL1, FPR1, GLT1D1, GMIP, GRAMD1A, HCK, IMPDH1, ITGAX, JUNB, KDM6B, LIMK2, LRFN1, LRG1, LRP10, MAPK3, MBOAT7, MIDN, MMP25, MSRB1, MTX1, MYO1F, NADK, NBEAL2, NCF4, NFAM1, PDLIM7, PFKFB4, PGD, PLIN3, PRKCD, PXN, RARA, RASGRP4, RGS19, S100A11, SBNO2, SEMA4A, SEMA4B, SHKBP1, SLC16A3, SLC19A1, SPI1, TECPR2, TFE3, TP53I11, TREML2, TSC22D4, VASP, WAS, XPO6, ZYX* **(Darked)**  *ABLIM3, ALOX12, ARHGAP6, CMTM5, CTDSPL, CTTN, EGF, EHD3, ELOVL7, ESAM, F13A1, GP1BA, GP6, GP9, ITGA2B, ITGB3, ITGB5, MAP1A, MGLL, PCSK6, PCYT1B, PEAR1, PF4, PTGS1, RHOBTB1, SELP, SPARC, TMEM40, TREML1, TSPAN9, TUBA8* **(Orange)**  *AURKB, CCNA2, CCNB1, CCNB2, CD38, CDC25A, CDC45, CDC6, CDCA2, CDCA5, CHPF, CLPTM1L, DERL3, DTL, FEN1, GLDC, GTSE1, HSP90B1, IGKC, IGLL5, IGLV3-1, IRF4, ITM2C, KIF2C, MCM10, MCM2, MCM4, MCM6, MZB1, NCAPG, NCAPH, ORC1, PDIA4, PDIA6, PLK1, POU2AF1, PRDX4, PYCR1, RPN1, RRM2, SEC61A1, SHCBP1, SLC1A4, SPAG5, TCF19, TNFRSF13B, TPX2, TRAM2, TXNDC11, TXNDC5, TYMS, ZWINT* **(Darkrange)** |


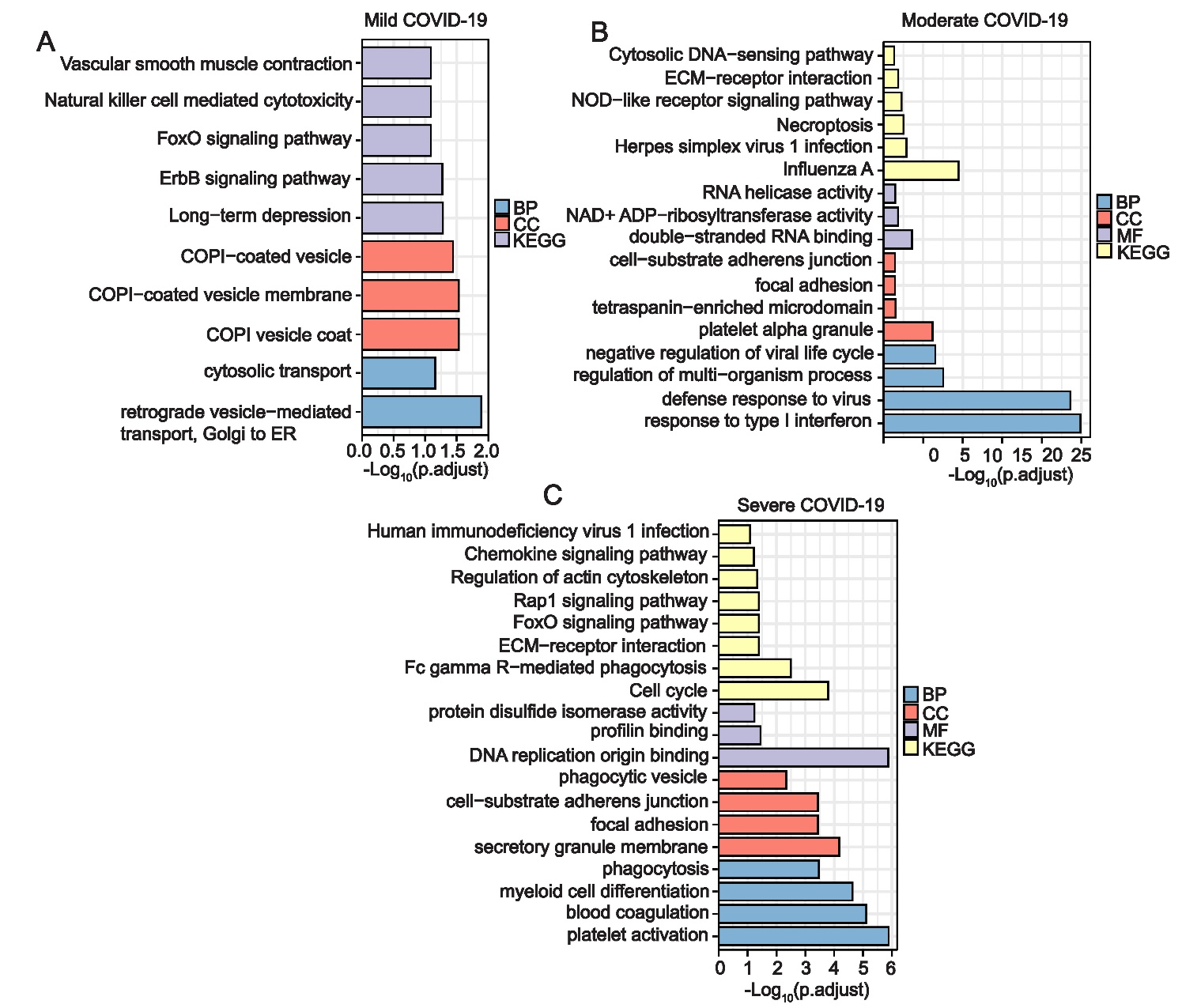


**Supplementary Figure 1.** Gene enrichment analysis of key genes in different status of COVID-19. Mild (**A**), Moderate (**B**), and Severe (**C**) enrichment results suggested that progressive severe immune and coagulation-related events were activated during COVID-19 progression.


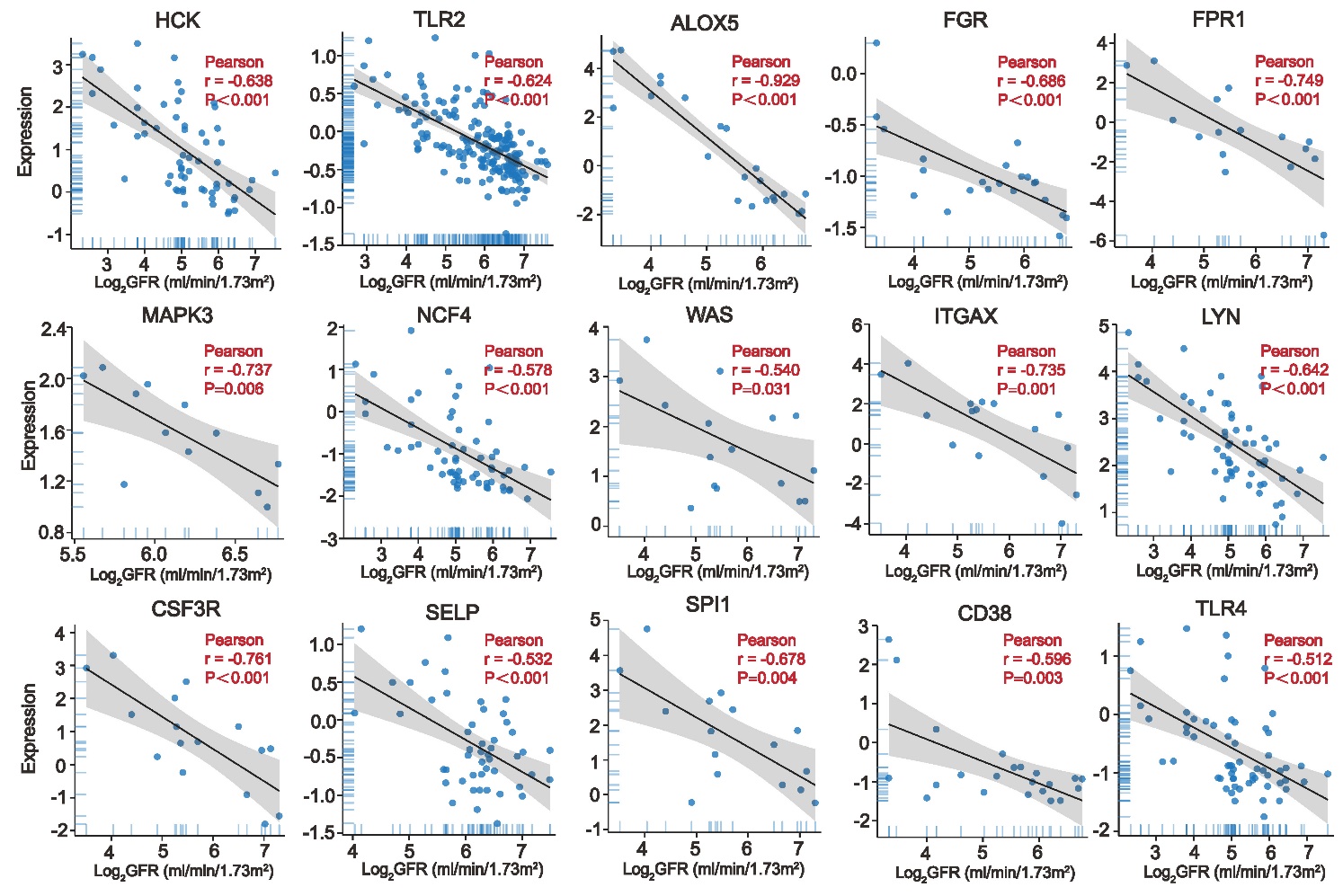


**Supplementary Figure S2 |** The correlation between PPI-hub genes and clinical indicators of kidney disease were explored in the Nephroseq database. Higher expression of PPI-hub genes was associated with lower glomerular filtration rate (GFR), suggesting that in COVID-19, these genes were correlated with poor kidney prognosis.
